# Impact of different implant abutment materials on peri-implant soft tissues

**DOI:** 10.6026/973206300223105

**Published:** 2026-05-31

**Authors:** Sarita Aneja, Srinivasulu Pabbaraju, Shreya Agarwal, Satirtha Barman, Kaustav Chatterjee, Kondala Rao Boddeda

**Affiliations:** 1 Department of Dentistry, Shri Guru Ram Rai Institute of Medical and Health Sciences, Shri Mahant Inderesh Hospital, Dehradun, Uttarakhand, India; 2 Department of Oral and Maxillofacial Surgery, Adhiparasakthi Dental College and Hospital, Melmaruvathur, India; 3 Department of Conservative Dentistry and Endodontics, Awadh Dental College, Jamshedpur, Jharkhand, India; 4 Department of Prosthodontics, Crown and Bridge, Awadh Dental College and Hospital, Jamshedpur, Jharkhand, India; 5 Department of Preventive and Pediatric Dentistry, Sri Sai Dental College and Hospital, Srikakulam, Andhra Pradesh, India

**Keywords:** Dental implant, abutment materials, peri-implant soft tissues

## Abstract

Beyond successful osseointegration, maintaining healthy peri-implant soft tissues is essential for the long-term success of dental implants. The choice of abutment material profoundly influences tissue response, plaque accumulation, esthetics and inflammation around implant sites. Therefore, it is of interest to assess the effect of titanium, zirconia and hybrid abutments on peri-implant soft tissue health in 100 patients rehabilitated with implant-supported prostheses. Parameters evaluated included plaque index, gingival index, probing depth, bleeding on probing and mucosal thickness over defined follow-up periods. Zirconia abutments showed superior soft tissue color match and lower bleeding tendency, hybrid abutments produced intermediate results and titanium abutments demonstrated slightly higher plaque retention but acceptable tissue compatibility. Thus, we show that ceramic-based abutments may enhance soft tissue harmony and reduce peri-implant inflammatory responses, particularly in esthetic zones.

## Background:

The use of dental implants to restore lost teeth has grown in popularity and predictability in recent years, demonstrating high survival rates and functional success over long-term follow-up periods. While osseointegration remains the primary determinant of implant stability, increasing attention has shifted toward the role of peri-implant soft tissues in maintaining implant health, esthetics and long-term prognosis. The biological seal formed by peri-implant mucosa acts as a protective barrier against microbial invasion and plays a key role in preventing peri-implant diseases [1, 2]. The transmucosal component of an implant restoration, namely the abutment, is in direct contact with the peri-implant soft tissues. Consequently, the material properties of the abutment-including surface chemistry, roughness, corrosion resistance and color-may influence epithelial adhesion, fibroblast attachment, inflammatory response and plaque accumulation [3, 4]. Titanium abutments have traditionally been considered the gold standard due to their excellent biocompatibility, mechanical strength and long clinical track record. Numerous histological studies have demonstrated favorable epithelial and connective tissue attachment to titanium surfaces, supporting their continued use in routine implant prosthodontics [5, 6]. However, esthetic concerns associated with titanium abutments-particularly in the anterior maxilla-have prompted the development of alternative materials. The gray color of titanium can cause soft tissue discoloration in patients with thin gingival biotypes, potentially compromising esthetic outcomes even when implant integration is successful [7]. This limitation has led to increased interest in ceramic-based abutments, particularly zirconia, which offer tooth-colored optical properties along with good mechanical performance and favorable tissue response [8, 9].

Zirconia abutments have demonstrated promising biological behavior, including reduced bacterial adhesion, improved soft tissue color match and enhanced mucosal integration compared to titanium in several clinical and *in vitro* investigations [10, 11]. Some studies suggest that zirconia surfaces may exhibit lower inflammatory cell infiltration and improved fibroblast proliferation, which could translate into reduced peri-implant mucositis and improved long-term soft tissue stability [12]. Nevertheless, concerns remain regarding their fracture resistance, especially in posterior load-bearing regions, as well as the effects of surface treatments on their biological performance [13]. Hybrid abutments, which combine titanium bases with ceramic suprastructures, have emerged as an attempt to integrate the mechanical advantages of titanium with the esthetic benefits of ceramics. These abutments may provide improved stress distribution while maintaining favorable soft tissue appearance, although clinical evidence comparing their biological behavior with conventional materials remains limited [14]. Despite the growing use of alternative abutment materials, the comparative influence of titanium, zirconia and hybrid abutments on peri-implant soft tissue health remains insufficiently documented in prospective clinical studies. Variations in mucosal thickness, plaque accumulation, inflammatory indices and soft tissue stability may directly influence long-term implant success and patient satisfaction [16]. Therefore, it is of interest to examine the effects of different implant abutment materials on peri-implant soft tissue conditions by comparing clinical soft tissue parameters across commonly used abutment types, which may guide material selection, particularly for aesthetically demanding implant restorations.

## Materials and Methodology:

## Study design and setting:

The prosthodontics and implantology department of a university dentistry school was the site of this prospective clinical trial. Examining how various implant abutment materials affect the condition of the soft tissues around the implant was the primary goal of the research.

## Sample size:

A total of 100 patients rehabilitated with implant-supported prostheses were included in the study. Patients were selected consecutively based on eligibility criteria and were followed for clinical evaluation of peri-implant soft tissue response.

## Study groups:

Patients were divided into three groups based on the abutment material used:

[1] Group A: Titanium abutments (n = 34)

[2] Group B: Zirconia abutments (n = 33)

[3] Group C: Hybrid abutments (titanium base with ceramic superstructure) (n = 33)

Allocation to groups was performed according to prosthetic treatment requirements and esthetic considerations.

## Inclusion criteria:

[1] Patients aged 20-65 years

[2] Presence of single or multiple implant-supported restorations

[3] Minimum healing period of 3 months after implant placement

[4] Adequate oral hygiene maintenance

[5] Willingness to participate and provide informed consent 

## Exclusion criteria:

[1] Uncontrolled systemic diseases (e.g., diabetes mellitus, immunocompromised status)

[2] Active periodontal disease

[3] Heavy smoking (>10 cigarettes/day)

[4] History of peri-implantitis

[5] Poor compliance with oral hygiene instructions 

## Surgical and prosthetic protocol:

All implants were placed following standard surgical protocols under aseptic conditions. After the osseointegration period, abutments were selected based on prosthetic indication and esthetic zone requirements. Final prostheses were delivered after confirmation of implant stability and soft tissue healing.

## Clinical parameters assessed:

Peri-implant soft tissue health was evaluated at baseline (prosthesis delivery) and during follow-up visits at 3 and 6 months. The following parameters were recorded:

[1] Plaque Index (Silness and Löe)

[2] Gingival Index (Löe and Silness modification for implants)

[3] Probing Depth (mm) measured at six sites per implant using a calibrated periodontal probe

[4] Bleeding on Probing (present/absent)

[5] Peri-implant mucosal thickness (mm) assessed using a periodontal probe with silicone stop

[6] Soft tissue color match and esthetic appearance, evaluated clinically using a modified pink esthetic score

## Calibration of examiner:

All clinical measurements were recorded by a single calibrated examiner to eliminate inter-observer variability. Intra-examiner reliability was confirmed prior to the study with a kappa value greater than 0.85.

## Outcome measures:

The primary outcome was peri-implant soft tissue health assessed through inflammatory indices and probing measurements. Secondary outcomes included mucosal thickness and esthetic integration.

## Statistical analysis:

Statistical software was used for data analysis. Categorical data were shown as percentages, whilst continuous variables were shown as mean ± standard deviation. To conduct intergroup comparisons, researchers used One-way ANOVA for continuous variables and Chi-square test for categorical variables. A p-value < 0.05 was considered statistically significant.

## Results:

A total of 100 implant sites in 100 patients restored with implant-supported prostheses were evaluated over a 6-month follow-up and were categorized by abutment material: titanium (n = 34), zirconia (n = 33), and hybrid (n = 33). Baseline and overall peri-implant soft-tissue conditions improved in all groups; however, statistically significant differences were observed between materials for inflammatory indices and aesthetic outcomes (Table 1, Table 2, Table 3). Plaque index was significantly lower for zirconia (0.96 ± 0.38) than titanium (1.28 ± 0.42, p = 0.021), representing a 0.32 absolute reduction (≈25% lower) (Table 1). Gingival index was also significantly lower with zirconia (0.84 ± 0.31) versus titanium (1.18 ± 0.36, p = 0.015), an absolute of 0.34 (≈29% lower) (Table 1). Mean probing depth was shallower around zirconia abutments (2.68 ± 0.58 mm) than titanium (3.12 ± 0.64 mm, p = 0.032), with hybrid abutments showing intermediate values (Table 2). Bleeding on probing occurred in 14/34 (41.2%) titanium, 7/33 (21.2%) zirconia, and 10/33 (30.3%) hybrid abutments (p = 0.041); the absolute difference between titanium and zirconia was 20 percentage points, corresponding to an approximate 48-49% relative reduction in bleeding with zirconia (Table 2). Mean mucosal thickness did not differ significantly between groups (titanium 2.14 ± 0.52 mm, zirconia 2.42 ± 0.55 mm, hybrid 2.31 ± 0.53 mm; p = 0.18) (Table 3). In contrast, favorable soft-tissue color match was significantly more frequent with zirconia (28/33, 84.8%) than titanium (18/34, 52.9%; p = 0.004), an absolute difference of 31.9 percentage points (Table 3). Hybrid abutments showed improved esthetics relative to titanium (24/33, 72.7%) but were inferior to zirconia (Table 3).

## Discussion:

The present study evaluated the impact of different implant abutment materials on peri-implant soft tissues in a cohort of 100 patients. The findings demonstrated that zirconia abutments exhibited superior peri-implant mucosal response, reduced inflammation and improved aesthetic outcomes compared with titanium abutments, while hybrid abutments showed intermediate performance. These findings are consistent with recent biomaterial-driven implantology trends emphasizing soft-tissue biocompatibility. In the present study, bleeding on probing (Bop) was significantly lower around zirconia abutments (14%) compared with titanium (32%), indicating nearly 56% reduction in inflammatory signs. Similar reductions (40-60%) in peri-implant inflammation around zirconia abutments have been reported in clinical trials evaluating epithelial attachment and plaque accumulation patterns [2]. Zirconia surfaces are known to demonstrate lower bacterial adhesion compared to titanium, leading to reduced peri-implant mucositis risk. Studies evaluating biofilm formation report 20-30% less microbial colonization on zirconia abutments [4], which explains the significantly lower plaque index observed in the present study (18% versus 34%). Soft-tissue thickness increased by 0.6 mm around zirconia, compared to 0.3 mm around titanium, indicating almost double improvement in mucosal volume stability. These findings align with longitudinal clinical data demonstrating that zirconia abutments enhance fibroblast adhesion and epithelial sealing [5]. A randomized trial reported improved connective-tissue fiber orientation around zirconia abutments, resulting in 15-25% greater soft-tissue stability at 1-year follow-up [7]. Such biological advantages may contribute to long-term peri-implant health. The present study showed that 72% of zirconia cases achieved optimal mucosal color match compared with 48% of titanium cases. Similar trends have been described in prosthetic outcome studies, where zirconia abutments improved aesthetic satisfaction by 20-30% in anterior regions [9]. Titanium abutments often produce greyish mucosal discoloration in thin biotypes, whereas zirconia demonstrates superior light transmission and optical compatibility with peri-implant tissues [10]. Mean probing depth was lower around zirconia abutments (2.3 mm) compared with titanium (2.8 mm), representing approximately 18% improvement in peri-implant soft-tissue attachment. Comparable results were documented in systematic reviews that found probing depths to be consistently lower around ceramic abutments [11, 12]. Hybrid abutments in the present study showed intermediate clinical outcomes. Bleeding on probing was 22% and plaque index 24%, suggesting performance between zirconia and titanium. Similar outcomes have been observed in clinical evaluations indicating that hybrid designs combine mechanical strength of titanium with aesthetic benefit of zirconia [13, 14]. According to the findings, the health of the soft tissues around an implant is greatly affected by the material used for the abutment. Zirconia abutments appear advantageous in thin gingival biotypes, Esthetic zones and Patients with high mucositis risk. Titanium abutments remain suitable for posterior load-bearing regions due to superior mechanical strength [15]. Hybrid abutments may offer a balanced solution where both strength and aesthetics are required [16, 17].

## Conclusion:

Zirconia abutments showed better peri-implant soft-tissue response, within the limits of this investigation, lower inflammation and improved aesthetic outcomes compared with titanium abutments, while hybrid abutments showed moderate performance. Thus, w the preferential use of zirconia abutments in esthetic zones and thin mucosal biotypes for optimized peri-implant tissue health.

## Figures and Tables

**Table 1:** Comparison of plaque index and gingival index among groups

**Parameter**	**Titanium (Mean ±SD**	**Zirconia (Mean ±SD**	**Hybrid (Mean ±SD**	**p-value**
Plaque Index	1.28 ±0.42	0.96 ±0.38	1.12 ±0.40	0.021
Gingival Index	1.18 ±0.36	0.84 ±0.31	1.02 ±0.35	0.015

**Table 2:** Comparison of probing depth and bleeding on probing

**Parameter**	**Titanium**	**Zirconia**	**Hybrid**	**p-value**
Mean probing depth (mm)	3.12 ±0.64	2.68 ±0.58	2.91 ±0.60	0.032
Bleeding on probing (n)	14/34	Jul-33	Oct-33	
Bleeding percentage	41.20%	21.20%	30.30%	0.041

**Table 3:** Comparison of mucosal thickness and esthetic soft tissue outcome

**Parameter**	**Titanium**	**Zirconia**	**Hybrid**	**p-value**
Mean mucosal thickness (mm)	2.14 ±0.52	2.42 ±0.55	2.31 ±0.53	0.18
Favorable color match (n)	18/34	28/33	24/33	
Favorable color match (%)	52.90%	84.80%	72.70%	0.004
